# What’s in a mechanism? Development of a key concept in realist evaluation

**DOI:** 10.1186/s13012-015-0237-x

**Published:** 2015-04-16

**Authors:** Sonia Michelle Dalkin, Joanne Greenhalgh, Diana Jones, Bill Cunningham, Monique Lhussier

**Affiliations:** University of Leeds, Leeds, UK; Northumbria University, Newcastle Upon Tyne, UK; Hadrian Primary Care Alliance, Newcastle Upon Tyne, UK

**Keywords:** Realist, Methodology, Palliative care, Realist evaluation, Realist synthesis

## Abstract

**Background:**

The idea that underlying, generative mechanisms give rise to causal regularities has become a guiding principle across many social and natural science disciplines. A specific form of this enquiry, realist evaluation is gaining momentum in the evaluation of complex social interventions. It focuses on ‘what works, how, in which conditions and for whom’ using context, mechanism and outcome configurations as opposed to asking whether an intervention ‘works’. Realist evaluation can be difficult to codify and requires considerable researcher reflection and creativity. As such there is often confusion when operationalising the method in practice. This article aims to clarify and further develop the concept of mechanism in realist evaluation and in doing so aid the learning of those operationalising the methodology.

**Discussion:**

Using a social science illustration, we argue that disaggregating the concept of mechanism into its constituent parts helps to understand the difference between the resources offered by the intervention and the ways in which this changes the reasoning of participants. This in turn helps to distinguish between a context and mechanism. The notion of mechanisms ‘firing’ in social science research is explored, with discussions surrounding how this may stifle researchers’ realist thinking. We underline the importance of conceptualising mechanisms as operating on a continuum, rather than as an ‘on/off’ switch.

**Summary:**

The discussions in this article will hopefully progress and operationalise realist methods. This development is likely to occur due to the infancy of the methodology and its recent increased profile and use in social science research. The arguments we present have been tested and are explained throughout the article using a social science illustration, evidencing their usability and value.

## Background

The idea that enquiry works by uncovering the underlying, generative mechanisms that give rise to causal regularities has become a guiding principle across many social and natural science disciplines. This article aims to provide a brief description of social mechanisms, mechanisms within evaluation and then specifically mechanisms in realist evaluation. The principles of Pawson and Tilley’s [1] conceptualisation of mechanism will then be discussed and operationalised through a reconceptualisation of the Context-Mechanism-Outcome configuration (CMOc) and an understanding of mechanisms on a continuum of activation.

### Much ado about mechanisms

#### Social mechanisms

One of the key tenets of realism is the very basic idea that observational evidence alone cannot establish causal uniformities between variables. Rather, it is necessary to explain why the relationships come about; it is necessary to establish what goes on in the system that connects its various inputs and outputs. In this manner, physicists are able fully to understand the relationship between the properties of a gas (as measured by the variables—pressure, temperature and volume) using knowledge about the kinetic action of the constituent molecules. In pharmacology, the term ‘mechanism of action’ refers to the specific biochemical interaction through which a drug substance acts on the body to generate its curative effect. Programme evaluators do not suppose that CCTV (the intervention) causes a fall in crime rates (the outcome). It does so, when it does so, by persuading potential perpetrators of increased risks of detection (the mechanism). In all cases, science delves into the ‘black box’. In all cases, the mechanism is what generates the observed relationship.

Whilst it is possible to recognise the affinities in explanatory structure across these examples, they also demonstrate that the action of the generative mechanisms is quite different, to such an extent indeed that that they defy a simple, unitary definition of their nature and content. Pawson expands on the applications of generative vs successive conceptualisations of causation elsewhere [2].

Readers of this journal will need no reminding that these paradigms have been debated for many years. Realists see physical and social reality as stratified and emergent. Things that cannot be cast as variables yet are vital to explanation (like kinetic forces, cultural norms and human interpretation or agency) are missing from correlational methods. Causal associations themselves are rarely universal; they are adaptive ‘demi-regularities’, which are always strongly influenced by setting and context. The original sources for these arguments may be found in Hesse [3], Harré [4], Pawson [2,5], Sayer [6,7], Bhaskar [8], Boudon [9] and Stinchcombe [10].

We acknowledge the further cleft between ‘critical realism’ and ‘scientific realism’. The writings of Bhaskar [8,11] and Pawson [2] serve as a reasonable proxy for these two schools. They differ on the matter of whether social science can create ‘closed system’ investigations. For Bhaskar, the closed system, experimental control available to the natural scientist is not achievable in social research because of ever-present emergence, that is to say the unique and unceasing human capacity to change the circumstances in which they live. As a ‘substitute’ for closed system empirical enquiry, he thus proposes the usage of abstract, *a priori* reasoning and the admission of a moral lens through which to critically evaluate human actions ([11], p. 64). Pawson, by contrast, argues much more pragmatically that neither physical science nor social science investigation depends on the achievement of closed systems ([5], p. 67). There are no crucial experiments (most especially Randomised Controlled Trials) which alone furnish us with social laws. But equally, natural science only ever makes slow and imperfect progress in gathering knowledge of the potentially infinite number of contingencies that can shape a physical system. Investigatory closure is always partial. Again, we are presented with rather different visions, the only contradiction occurring when an investigation claims to be *both* normative and scientific.

For Archer [12], collective, constrained decision making is the underlying mechanism that creates all social outcomes. Society is made by but never under the control of human intentions. At any given time, peoples’ choices are conditioned by pre-existing social structures and organisations. We are thus externally constrained in our actions but always part of human agency is the choice to attempt to change the initial conditions that bear down on us. These adaptive choices, over time, go on to mould novel structures and changed institutions. Collectively, our present decisions congregate to form new systems, which in their own turn, constrain and enable the choices of the next generation. Society is thus patterned and re-patterned by wilful action, but as Archer reminds us, the causal outcomes never conform to anyone’s wishes—even the most powerful.

Most realists would affirm this broad account of the mechanisms of social change, where structures shape actions, which shape structure, which shape actions, and so on. There are, however, some significant differences in where they locate the precise locus of that change. For Bhaskar [8], causal mechanisms sit primarily within the structural component of the social world. They reside in the power and resources that lie with the great institutional forms of society. For other realists, such as Pawson and Tilley [1], mechanisms are identified at the level of human reasoning. Thus, mechanisms can have different meanings depending on the scope of the intended explanation. Structural mechanisms come to the fore if the social scientist is attempting to explain large-scale social transformations. If, however, the researcher is attempting to discover whether a particular fitness programme creates healthier participants, it can be assumed that key outcomes will result from the reasoning and responses of the participants.

#### Mechanisms in evaluation

This brings us to a consideration of mechanisms in evaluation research; here the focus is on developing an explanation of how a particular programme works through changing the reasoning and responses of participants to bring about a set of intended outcomes. There have been a number of different conceptualisations of mechanism within evaluation. Chen and Rossi [13] were among the first researchers to use the term ‘mechanism’ and highlight its significance in theory-driven evaluation [14]. In 2005, Chen [15] broadened our understanding of causal mechanisms by identifying two types: mediating and moderating. He defines these as follows:

*“A mediating causal mechanism is a component of a program that intervenes in the relationship between two other components . . . [while] the second type of causal mechanism—moderating—represents a relationship between program components that is enabled, or conditioned, by a third factor.”* (pp. 240–241)

Weiss [16] also reflects on mechanisms, in terms of programme theory. She states that it is important to understand the difference between implementation theory and programme theory. The earlier can be conceptualised as a logic model, whereas the latter:

*“. . . deals with the mechanisms that intervene between the delivery of program service and the occurrence of outcomes of interest. It focuses on participants’ responses to program service. The mechanism of change is not the program service per se but the response that the activities generate.”* (p. 46)

As Weiss [16] states, mechanisms are not the programme service but the response it triggers from stakeholders and resulting outcome. For example, Vassilev et al.’s [17] metasynthesis investigated how social networks can make a considerable contribution to improving health outcomes for people with long-term conditions (specifically, type 2 diabetes). They identified three themes which translated into three ‘network mechanisms’: *network navigation* (identifying and connecting with relevant existing resources in a network), *negotiation within networks* (re-shaping relationships, roles, expectations, means of engagement and communication between network members) and *collective efficacy* (developing a shared perception and capacity to successfully perform behaviour through shared effort, beliefs, influence, perseverance, and objectives). The authors highlight not only resources in these mechanisms but also reasoning; these mechanisms convey the close interdependence between social and psychological processes in long-term conditions management. Furthermore, these network mechanisms are subject to context, as the authors state:

*“they are shaped by the environments in which they take place which can be enabling or disabling depending on the capacities they offer for carrying out illness management work and supporting behaviours beneficial for people’s health.”* (p. 10)

Despite the many different conceptualisations, e.g. [9,13-16,18], and applications of mechanisms, e.g. [17,19,20], most in some way have been influenced by the critical realism and scientific realism accounts of causation, e.g. [1,21,22], discussed above. In these schools of thought, mechanisms are usually hidden, sensitive to variations in context and generate outcomes. As Astbury and Leeuw [14] state, mechanisms in realism are:

*“underlying entities, processes, or structures which operate in particular contexts to generate outcomes of interest.”* (p. 368)

We survey this broader terrain as a prelude to focussing on the more specific version of mechanism thinking referred to by Pawson and Tilley that has come to play a key role in the evaluation of social interventions, namely realist evaluation [1], which is the main focus of this article.

#### Mechanisms in realist evaluation

Within the scientific realism approach, Pawson and Tilley [1] have provided their own conceptualisation of mechanisms; mechanisms are a combination of resources offered by the social programme under study and stakeholders’ reasoning in response [1]. They state that mechanisms will only activate in the right conditions, providing a context + mechanism = outcome formula as a guiding principle to realist enquiry [1]. This article sits within the empirical application of realism in the form of realist evaluation and the usage of mechanisms therein. In particular, we make a case for the explicit disaggregation of resources and reasoning in implementation endeavours, to which task we now turn.

The units of analysis within realist evaluation are programme theories—the ideas and assumptions underlying how, why and in what circumstances complex social interventions work. Many readers will by now be very familiar with programme theories expressed as CMOc and with the fact that data collection and analysis in realist evaluation centres on the process of developing, testing and refining CMOc. In the next section of the paper, we propose a development of this formula, which aims to facilitate the study of implementation processes and interventions.

### A social science illustrative case study

In order to illustrate our argument in this article and maximise explanatory reach, we draw on empirical data from our realist evaluation of a palliative care Integrated Care Pathway (ICP). The ICP aimed to improve the co-ordination of care for people in the final year of life by identifying individuals approaching end-of-life, assessing and agreeing how needs and preferences of patients could be met, providing support for families and carers and using Advance Care Planning (ACP) to manage the patients’ final illness in order to achieve a ‘good’ (preference based) death. The ICP comprised a variety of interventions including palliative care registrations, ACP and multidisciplinary team meetings in order to anticipate and plan care for patients with palliative care needs. We evaluated the implementation of the ICP across 14 GP practices in one UK locality using realist evaluation. Five initial programme theories, generated from immersion in the field and literature on ICPs, were tested: (1) the embeddedness of the ICP into GP practices, (2) the registration of palliative care patients, (3) preference discussions and ACP, (4) facilitating difficult conversations and (5) facilitating home deaths. The five refined programme theories were combined to create one overall programme theory of the whole ICP. This encapsulated the ICP as a translational tool of national policy drivers (such as shared decision making, patient-centred care and proactive care) into local practice.

Using realist evaluation to shed light on how such a complex intervention could work in practice made intuitive sense but proved not to be without operational challenges. These have been echoed by other realist researchers [23-25] and have prompted the writing of this paper.

This paper has two main aims:

- To make a case for the explicit disaggregation of resources and reasoning within mechanisms;
- To reiterate the need for nuance in considering whether mechanisms fire in a dual on/off mode.

## Discussion

### Disaggregating mechanisms into resources and reasoning

#### 1 The concern

Realists posit that exposing not only the mechanisms of change in an intervention but more importantly their relationship to the context of their implementation is key to the evaluation of complex programmes [20,26]. However, deciding whether aspects within an intervention implementation process in a realist project contribute contextually or mechanistically to the overall explanatory endeavour has become the realist researcher’s quandary [14,23,27]. Like these authors, we encountered challenges in distinguishing between context and mechanism in our evaluation of the ICP and were cognisant of the need not to conflate programme strategy (the intervention) with mechanism. We concur with Jagosh et al. [23], who note how it is not always as straightforward as might be assumed to map the complexities of the transformation process and the multiple systems within which it operates onto the C + M = O formula. Arguably, outcomes can be identified with most ease; they are observed or measured or at least aimed at with a degree of clarity. Although the distinction between resources and reasoning is used in Pawson and Tilley’s seminal work [1], their relative importance in understanding mechanisms is often understated. Consequently, researchers often emphasise one at the expense of the other, under the banner of mechanism [25]. To address this, we offer the solution below.

#### 2 Our way forward

Building on the original work of Pawson and Tilley [1], we would like to propose an alternative operationalisation of the CMOc formula:

Intervention resources are introduced in a context, in a way that enhances a change in reasoning. This alters the behaviour of participants, which leads to outcomes.

The revised formula therefore reads:

$$ \mathbf{M}\left(\mathbf{Resources}\right)+\mathbf{C}\to \mathbf{M}\left(\mathbf{Reasoning}\right)\kern0.7em =\kern0.7em \mathbf{O} $$

**Figure 1:**
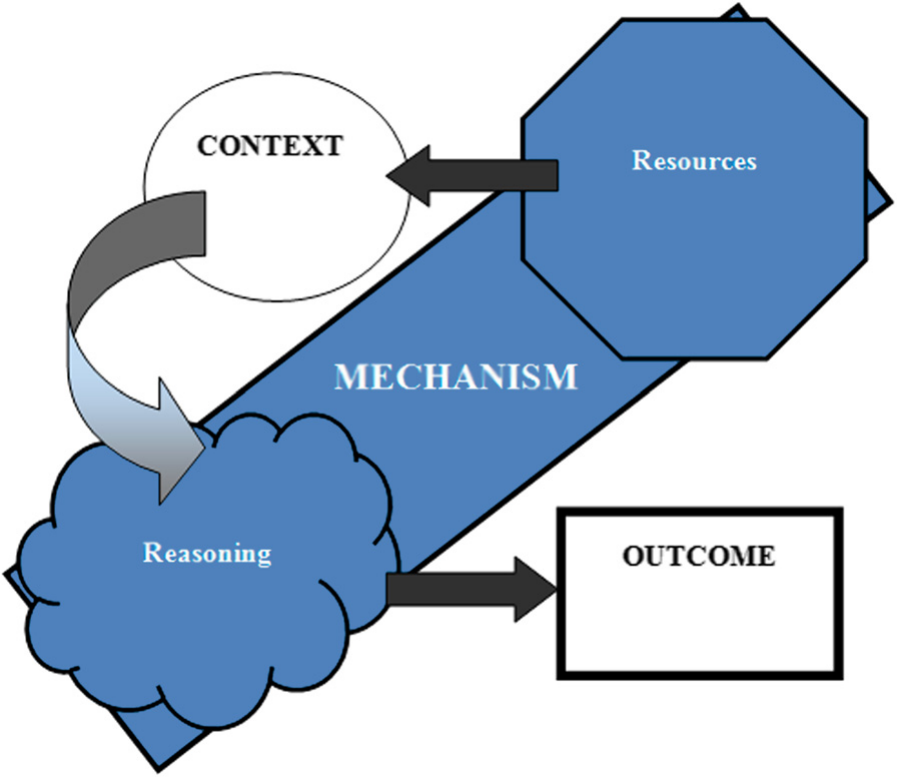
A CMOc framework.

Resources and reasoning are mutually constitutive of a mechanism, but explicitly disaggregating them can help operationalise the difference between a mechanism and a context. Although resource and reasoning are made explicit in the seminal work of Pawson and Tilley [1], they have often not been referred to explicitly in subsequent research. In our own study, through using this formula, it became clearer whether data contributed contextually or mechanistically, as we could identify mechanism components (resource and reasoning) which are different to contexts. Figure 1 illustrates how we have presented the new formula diagrammatically in the ICP study. Through trial and error, it became clear that the original formula could be built upon, hence the new formula which disaggregates resource and reasoning, placing ‘context’ in between. However, this is not to be confused with just using resources without reasoning—they must always come as a pair. It is important to note here that this new formula is only an extension of the original heuristic developed by Pawson and Tilley [1]. This new formula does not aim to re-draw the full sequence of causation but to modify the basic heuristic to aid operationalisation of realist approaches.

Differentiating between resource (the component introduced in a context) and reasoning therefore helps distinguish between relevant context and mechanism. Identifying the resource is contingent on the purpose of the study, and identifying the reasoning avoids the issue of conflating programme strategy (resource) with mechanism.

#### 3 The social science illustration

**Figure 2:**
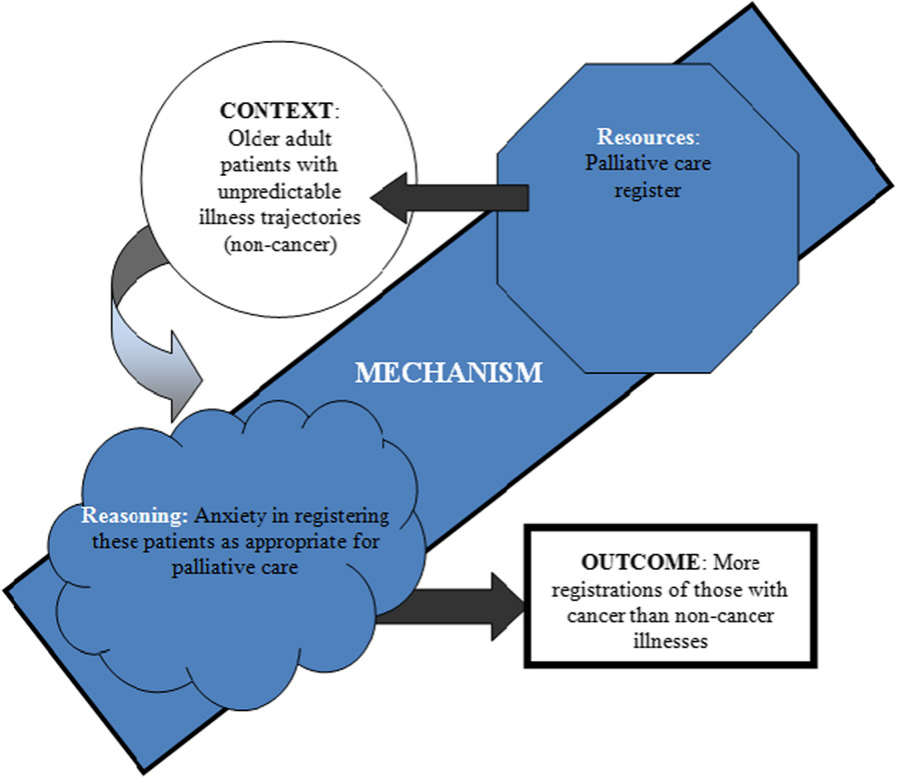
Refined CMOc for patients in care homes receiving the ICP.

In the palliative care ICP study, an outcome pattern was observed that practices identified and placed fewer palliative patients with non-cancer illnesses on their palliative care registers, in comparison to those with cancer illnesses. This was common across all 14 practices studied and was particularly noticeable for patients residing in care homes, where many older adults have non-cancer illnesses. Patients with non-cancer illnesses have unpredictable illness trajectories, meaning that registering this patient group is challenging for health care professionals, as a period of significant decline can be followed by substantial improvement, despite a downward trend in wellness [28,29]. Comparatively, this is not the case with cancer diagnoses as often there is a specific diagnosis and steady illness trajectory. We aimed to generate a CMOc to explain why there were less palliative care registrations of patients with non-cancer illnesses than cancer patients (outcome). In attempting to formulate the configuration, we were uncertain whether the context was the unpredictable illness trajectories of older adults without a cancer diagnosis, or care homes in general or the palliative care register being difficult to use with non-cancer patients. Breaking down the C + M = O formula to include resource and reasoning using the new formula, M (resource) + C → M (reasoning) = O, helped in deciphering the context from the mechanism. The use of the new formula diagram (Figure 2) also helped in configuring the whole CMOc. Figure 2 displays the novel way in which the new formula should be represented diagrammatically. Through using the new formula and associated diagram, it became clear that the resource was the palliative care register which, when used with older adults who had unpredictable illness trajectories (context), resulted in anxiety in registering these patients (reasoning), which meant that less older patients in care homes were registered (outcome) (Figure 2). Through understanding that resources were introduced into pre-existing contexts in a way that altered the participants’ reasoning, it becomes easier to explain the differential registration numbers (outcome).

Disaggregating resources and reasoning encourages researchers to consider both concepts, rather than privileging one at the expense of the other and will contribute significantly to the explanatory endeavour of the realist researcher. It is important to understand the new formula (M (Resource) + C → M (Reasoning) = O) highlights that resources must be introduced into a pre-existing context, which in collaboration induces an individual’s reasoning, leading to an outcome. Distinguishing the resources that are introduced into contexts from the reasoning this generates can provide both an operational and a conceptual clarification of mechanism. It can enable researchers to clearly understand the role of context in triggering mechanisms, thus developing their explanation of how interventions work. We now turn to interrogate the notion of mechanisms being ‘triggered’ in the next section of the paper.

### A case for continuums of activation in reasoning

#### 1 The concern

A separate but related difficulty encountered when using mechanisms in social science research is the notion that mechanisms are often said to ‘fire’, ‘trigger’ or ‘modify’ in context to create an outcome [1,30-32]. Pawson and Tilley [1] use the much referenced gun powder analogy to explain this. When a spark is introduced to gun powder, the chemical composition of gun powder (mechanism) results in an explosion (outcome). However, there are no explosions if the context is not right—damp conditions, insufficient powder, not adequately compact, no oxygen present, duration of heat applied is too short (context). Thus it purports that causal outcomes follow from mechanisms acting in contexts; this is the base from which all realist explanation builds. Most complex social interventions involve stakeholders’ volition (reasoning). As Pawson [33] states, “much more than in any other type of social programme, interpersonal relationships between stakeholders embody the intervention” [33]. We found it difficult to apply the firing analogy to interventions where human volition is entwined in the intervention. Reasoning in these cases is rarely activated via an on/off switch, triggered in favourable contextual conditions. Instead, activation operates along a continuum similar to the light created by a ‘dimmer switch’, where intensity varies in line with an ever evolving context. Our experience suggests that researchers are often enabled to develop their realist thinking further when this myth of on/off reasoning is dispelled. The metaphor of the dimmer switch accommodates the activation of new volition as well as the idea of continuums of activation.

#### 2 Our way forward

Conceptualising volition as happening in a binary ‘firing’/‘not firing’ fashion masks a continuum of activation which can have more explanatory value in understanding how interventions work. There are varying degrees to which an individual can feel confident, angry or mistrustful, leading in turn to a gradation of outcomes.

#### 3 The social science illustration

In our evaluation of the ICP, we observed that the volition of health care professionals was always on a continuum. Health care professionals felt anxious when registering older adults with an illness other than cancer, as the trajectory of such illnesses is so unpredictable (Figure 2). Health care professionals could not predict patients’ decline, did not wish to over populate their palliative care registers and were worried about registering patients who seemed relatively well but could decline quickly. Furthermore, once a decline in health begins in older adults with non-cancer illnesses, it can be very rapid and thus end-of-life care is implemented quickly and is often unplanned, which can result in a death that does not adhere to patient preferences. The anxiety of health care professionals working with palliative non-cancer patients was evident, yet this anxiety did not switch on and off, it developed over time, as patients’ illnesses progressed. It also differed between health care professionals; those with more experience of working with patients with non-cancer disease had less anxiety about registering them. Thus the reasoning of having anxiety was on a continuum for health care professionals using the palliative care register. There is a variation in the amount of anxiety a health care professional will feel when registering a patient with a non-cancer illness, it is not dichotomised; the degree to which this is felt is combined with a facilitative context and appropriate resource. This should lead to a more appropriate use of the palliative care register.

## Summary

This paper aimed to help the operationalisation of the C + M = O formula, through (1) a disaggregation of the mechanism resource and mechanism reasoning and (2) a conceptualisation of activation continuums, rather than a binary trigger. The solutions proposed in this article will enable a clearer application of realist evaluation to understanding how complex interventions are implemented. We have already found some evidence to support this argument by applying it in our own teaching and workshops. For example, the ‘workability’ of this framework has been tested with researchers at the beginning of their realist journey in a realist summer school at the Centre for Advancement in Realist Evaluation and Synthesis (CARES), University of Liverpool. Course participants found it useful to guide their realist learning, understand the method further and clarify the differences between mechanism and context, and resources and reasoning.

We hope that this article furthers the discussions on the operationalisation of realist theory development in a way that, in particular, helps novice realist researchers to embrace and in turn develop the methodology. The authors would welcome testing of the methodological refinements discussed throughout this article by other researchers across a wide range of fields, with such testing aiding further developments.

## Abbreviation

CMOc: Context Mechanism Outcome configuration
